# Short-term forecasting approach of single well production based on multi-intelligent agent hybrid model

**DOI:** 10.1371/journal.pone.0301349

**Published:** 2024-04-17

**Authors:** Hua Yan, Ming Liu, Bin Yang, Yang Yang, Hu Ni, Haoyu Wang

**Affiliations:** 1 School of Energy and Power Engineering, University of Shanghai for Science and Technology, Shanghai, China; 2 Shanghai Key Laboratory of Multiphase Flow and Heat Transfer in Power Engineering, University of Shanghai for Science and Technology, Shanghai, China; 3 Petroleum Engineering Technology Research Institute of Shengli Oilfield Company, SINOPEC, Dongying, China; Newcastle University, UNITED KINGDOM

## Abstract

The short-term prediction of single well production can provide direct data support for timely guiding the optimization and adjustment of oil well production parameters and studying and judging oil well production conditions. In view of the coupling effect of complex factors on the daily output of a single well, a short-term prediction method based on a multi-agent hybrid model is proposed, and a short-term prediction process of single well output is constructed. First, CEEMDAN method is used to decompose and reconstruct the original data set, and the sliding window method is used to compose the data set with the obtained components. Features of components by decomposition are described as feature vectors based on values of fuzzy entropy and autocorrelation coefficient, through which those components are divided into two groups using cluster algorithm for prediction with two sub models. Optimized online sequential extreme learning machine and the deep learning model based on encoder-decoder structure using self-attention are developed as sub models to predict the grouped data, and the final predicted production comes from the sum of prediction values by sub models. The validity of this method for short-term production prediction of single well daily oil production is verified. The statistical value of data deviation and statistical test methods are introduced as the basis for comparative evaluation, and comparative models are used as the reference model to evaluate the prediction effect of the above multi-agent hybrid model. Results indicated that the proposed hybrid model has performed better with MAE value of 0.0935, 0.0694 and 0.0593 in three cases, respectively. By comparison, the short-term prediction method of single well production based on multi-agent hybrid model has considerably improved the statistical value of prediction deviation of selected oil well data in different periods. Through statistical test, the multi-agent hybrid model is superior to the comparative models. Therefore, the short-term prediction method of single well production based on a multi-agent hybrid model can effectively optimize oilfield production parameters and study and judge oil well production conditions.

## Introduction

In the process of oilfield production, single well production is one of the essential indicators of oil well productivity, which has crucial reference value for understanding the dynamic situation of reservoir blocks and evaluating the development potential of oilwell blocks. The short-term prediction of single well production can provide direct data support for timely guiding the optimization and adjustment of oilfield production parameters and studying and judging oil well production [1]. The primary objective of this study is to address the issue of accurate prediction of oil production, as the non-stationarity and volatility of historical oil production data, subject to various external factors, pose significant challenges for prediction studies. Therefore, accurate oil production forecasting can estimate oil capacity, discern the working status of oil wells, and provide reliable supporting data for oilfield production planning, thereby serving the construction of smart oilfields.

The single well production data of the oilfield is affected by various uncertain factors such as reservoir porosity, permeability, saturation, oil-water viscosity ratio of crude oil, percolation mechanics, and so on, with great randomness and volatility [2]. The prediction model of single well production data based on the mechanism is usually established based on the formation model, oil production theoretical model and physical oil parameters. The oil production is predicted according to various state parameters collected. The prediction accuracy of such methods is greatly affected by the uncertainty and complexity of the parameters in the theoretical models, which requires a large number of monitoring data from sensors and test instruments or simulation data obtained through theoretical numerical simulations. It needs to match the whole oilfield d production technology and process for analysis. Not only is it difficult to obtain accurate short-term predictions, but also for practical applications [3–6].

With the promotion of smart oilfield construction, the automation and digitization of oilfield production have significantly been developed, enabling the data island formed by a large number of oilfield production data, which provides a strong guarantee for data-driven prediction modeling [4]. The data-driven prediction model learns the cohesive correlation characteristics of the dataset through the oil well production sequence data, overcomes the difficulty of traditional methods in solving complex physical processes efficiently, and can effectively predict the oil production of oilwells [7]. At present, data-driven models are divided into three categories: mathematical statistics model, artificial intelligence model, and hybrid model.

Mathematical statistical prediction models for oil production usually use mathematical statistical models, such as Decline Curve Analysis (DCA). Auto Regression (AR), Moving Average (MA), Auto Regression Moving Average (ARMA)and autoregressive integrated moving average (ARIMA), to analyze historical data such as production and environment and predict oil field production [8, 9]. Such models are mainly used for long-term prediction through data trends. It is difficult to capture the data characteristics with large short-term fluctuations under multiple factors. Wang et al. [10] combined ARIMA and grey model to build a shale oil production prediction method, and used quarterly production data to verify the prediction performance. The results showed the method’s superior prediction performance and accuracy in the long-term trend.

The artificial intelligence prediction model represented by machine learning has developed rapidly in recent years [11]. This kind of method does not need additional reservoir characteristics and production parameters. It can obtain data structure information from previous data and establish a black box data model from the front performance of the research object to the results to establish a model for the research object. Various specific internal structures and data flow processing methods of machine learning models can mine the potential characteristics of data and the correlation between forward and backward. The established association model can efficiently, accurately, and reliably map the input state of data to the output mode. More common machine learning prediction models include Decision Tree (DT), Random Forest (RF) [12], support vector machine regression (SVMR) [13], Least Square Support Vector Regression (LSSVR) [14], Extreme Learning Machine (ELM) [15], etc. Chahar et al. [16] respectively used RF, Gradient Boosting Regressor (GBR), and Artificial Neural Networks (ANNs) [17] to predict and analyze oil and gas production, and concluded that RF and ANNs had outstanding prediction performance on different datasets.

Although the above Machine Learning (ML) model has the advantages of good prediction performance and fast training speed, it only regards the sequence data as the data dimension, does not care about the internal cause-and-effect relationship between data sequences, and only establishes the correspondence between data input and output at a superficial level. The rapid development and deepening application of machine learning have extensively promoted the development of neural networks and Deep Learning (DL) theory. Various application-specific network layers have also been bred, such as Recurrent Neural Network (RNN) [18], which are good at processing time series data, as well as Convolutional Neural Network (ONN) [19]. Meanwhile, DL constructed with multiple neural networks has been gradually applied to predict oil and gas production with their strong predictive generalization and high prediction accuracy. Sagheer et al. and Al Shabandar et al. carried out oil production prediction research on stacking of Long Short-Term Memory (LSTM) network and Gated Recurrent Unit (GRU), respectively [20, 21]. It has become a research trend in the field of time series prediction that the prediction model combined with intelligent model and data decomposition can reduce the difficulty of data feature recognition. Liu et al. [22] employed artificial neural networks and machine learning, combined data preprocessing operation, to build a predictive model for oil production and obtain high-precision prediction results, which reflected that the prediction effect of the single combination prediction model established by the combination of a single model and decomposition technology was better than that of the single model.

In view of the small training loss and low convergence of the single combination prediction model in the specific training process, the multi-combination model composed of multiple models and decomposition technology can effectively solve the above problems and further improve the model’s prediction performance [23, 24]. Altan et al. [25] utilized the Grey Wolf Optimizer (GWO) to optimize Long Short-Term Memory (LSTM) and combined it with data decomposition techniques to construct a hybrid model for wind speed prediction. Liu et al. [26] proposed a multi-step wind speed prediction model based on combining long-term and short-term memory networks, extreme learning machine, and decomposition. The results showed that the multi-step combination model had better prediction accuracy than the single-combination model. Coşkun et al. [27] present a hybrid method that leverages the strengths of data decomposition techniques and LSTM individually to investigate prediction of the standardized precipitation index, and demonstrates the advantages of hybrid method in forecasting through validation. Similar to wind speed data, daily production data from oil wells have strong volatility and poor stability properties, hence multi-combination models can provide a more accurate prediction method for oilwell production.

Therefore, a short-term prediction method is proposed for oil production of a single well by combining data decomposition and multi-combination model in this paper. This method first decomposes and reconstructs the one-dimensional time series data of daily oil production of a single well. The reconstructed components are grouped after clustering and entropy analysis. After grouping and fusion, the two types of intelligent models are used for grouping prediction. Finally, the final oil production prediction result is formed by superposition, which provides data support for timely guiding the optimization and adjustment of oilfield production parameters and studying and judging the production conditions of oil wells.

## Methodology

### Overall process of prediction

Considering that the short-term time series data of daily oil production of a single well are comprehensively affected by many factors, such as porosity, permeability, oil-water saturation, oil-water viscosity ratio of crude oil and inter-well connectivity, showing greater randomness and volatility, and the internal mechanism of the data is relatively complex, it is difficult to accurately predict using a single model. In this regard, combined with data decomposition and a multi-agent combination model, this paper proposes a short-term prediction method for oil production of a single well in the oilfield, and constructs the short-term prediction process of oil production of a single well in the oilfield as shown in S1 Fig, including data decomposition, dataset generation, component aggregation, model allocation, and prediction fusion.

Step 1. Data decomposition.

Raw data was decomposed and reconstructed by Complete Ensemble Empirical Mode Decomposition with Adaptive Noise Analysis (CEEMDAN) to form Intrinsic Mode Function (IMF)s and the residual. After calculating the Fuzzy Entropy (FE value and autocorrelation coefficient (AC) value of each IMFs without the residual. the two values form a vector as the corresponding IMF feature vector.

Step 2. Dataset generation.

The raw data of daily well production and the IMFs obtained after decomposition are one-dimensional sequential data, which cannot be directly adopted for model training and learning.

Step 3. Data distribution.

IMFs are obtained after decomposing the training data are kept, while the residual components are discarded. Firstly, FE value and autocorrelation of each IMF are calculated, which are then normalized to the range of 0,1 in order to form the data vector space. Feature vectors in the vectors space above are clustered into two groups, after which the group including the vector of minimal vector Euclidean Distance (ED) [28] value is signed as "ML-group" while the other is signed as "ELM-group". By the procedure described above, the IMFs are reasonably distributed.

Step 4. Data prediction.

The data output in step (3) is grouped and predicted. Two sub-prediction models. machine learning and deep learning, are used to form a multi-agent combination model. The machine learning model is an Online Sequential Extreme Learning Machine (OSELM) model based on the Whale Optimization Algorithm (WOA)The deep learning model is a convolutional bidirectional gated cyclic network model with self-attention mechanism (ED-CNN-BiGRU-Att) based on Encoder-Decoder architecture.

Step 5. Fusion of predicted results.

The two values predicted in the above step are again added together to obtain the final well predicted yield.

### Data decomposition algorithm

CEEMDAN is used to decompose the training set, to reconstruct and form multiple groups of IMFs and residuals, obtain data components under different frequency characteristics, and reduce the complexity of data influencing factors, which is used to decompose the training set as data decomposition approach in this paper, because the result decomposed by CEEMDAN can ameliorate the modal aliasing phenomenon [29], and the completeness of the data reconstruction is better via adding adaptive noise as well. The basic principles of the CEEMDAN algorithm are briefly described as follows.

Step 1: Signal *T*(*t*) = *T*_0_(*t*)+*a*_0_*n*^i^(*t*) is constructed from the original signal *T*_0_(*t*) by adding noise, where *a*_0_ refers signal-to-noise ratio control coefficient, and *n*^i^(*t*) (*i* = 1,2, …, *N*) is the white Gaussian noise. In the first phase, the newly generated data signal needs to be initially decomposed by EMD to obtain $IMF_{1}^{i}(t)$ via (1). after which the first-order residual data signal is given by *T*_1_(*t*) = *T*(*t*)-*IMF*_1_(*t*).


$$IMF_{n}(t)=\frac{1}{N}\sum_{1}^{N}IMF_{n}^{i}(t)$$ (1)

Step 2: A new signal *T*_*2*_(*t*) = *T*_1_(*t*)+*a*_1_*E*_*1*_*[n*^i^(*t*)] is constructed by adding new noise signal. Here, *E*_*k*_[*] indicates the function that generates the first phase. And then, repeating calculation by Step 1, *IMF*_2_(*t*) and *T*_3_(*t*) are both achieved.

Step 3: Following steps 1 and 2, multiple mode components, as well as residual signals, can be obtained with the expression as follows:

$$T_{0}(t)=\sum_{k=1}^{K}IMF_{k}(t)-R(t)$$ (2)

### Dataset generation algorithm

As shown in S2 Fig, the sliding window segmentation method is used to divide the dataset into several sample sets for data generation in this paper. This method takes *W* data as the input sequence of the model from the *N*th position of the data set, and then takes 1 data value as the model’s output to form the data set of one-step prediction. *N* counts from 1 until all the training sets are segmented, and *W* represents the input length of a group of data [30]. For example, starting from the dataset, a continuous sequence of data with a length of *W* from the first data point are selected as the first input for the training set, denoted as *X*_1_ = (*x*_1_, *x*_2_, …, *x*_*w*_). At this point, the output of the training set is *Y*_1_ = *x*_*w*+1_. Subsequently, the input to the second training set is denoted as *X*_2_ = (*x*_2_, *x*_3_,…, *x*_*w*+1_) and its corresponding output is denoted as *Y*_2_ = *x*_*w*+2_. This process continues until all data has been allocated.

### Data distribution process

Since entropy is a measure of the complexity and chaos of sequence data, the greater its value indicates that, the more frequent the data sequence contains, the greater the chaos of the sequence [31], which can accurately reflect the complexity of daily oil production of a single well. Given a sequence *x*(*i*), *i* = 1 to N, the sequences $X_{i}^{m}$, *i* = 1 to *N*-*m*+1, is established as given below.

$$X_{i}^{m}=\{x(i),x(i+1),\ldots ,x(i+m-1)\}-x_{0}(i)$$ (3)

where *m* refers to the length of the sequences, *x*_0_(*i*) is a base line, and $D_{\left\{ij\right\}}^{m}(n,r)$ denotes the similarity degree using fuzzy membership function $\mu (d_{\left\{ij\right\}}^{m},n,r)$ for the vector $X_{i}^{m}$ and $X_{j}^{m}$.

$$\left\{ \begin{array}{l} D_{ij}^{m}(n,r)=\mu (d_{ij}^{m},n,r)\\ \mu (d_{ij}^{m},n,r)=e^{\frac{-{\left(d_{ij}^{m}\right)}^{n}}{r}} \end{array}\right.$$ (4)

where *n* and *r* are width of the exponential function and predefined gradient, $d_{ij}^{m}$. denotes the maximum absolute difference between $X_{i}^{m}$. and $X_{j}^{m}$, and the function is defined as follow:

$$\phi^{m}(n,r)=\frac{1}{N-m}\sum_{i=1}^{N-m}\left(\frac{1}{N-m-1}\sum_{j=1,j\neq i}^{N-m}D_{ij}^{m}\right)$$ (5)

$X_{i}^{m+1}$. as the sequences is generated by *m* = *m*+1, and *φ*^*m*^(*n*,*r*) is constructed afterwards. Finally, the fuzzy entropy is calculated as given below:

$$FE(m,n,r,N)=\frac{\phi^{m}(n,r)}{\phi^{m+1}(n,r)}$$ (6)

In addition, autocorrelation coefficient, as another quantitative metric, is calculated as follow:

$$AC=\sum_{i=1}^{n-r}\frac{\left(x_{i}-\mu )(x_{i+r}-\mu \right)}{\sum_{i=1}^{n}{\left(x_{i}-\mu \right)}^{2}}$$ (7)

The IMFs feature vectors, constructed as (*V*_*FE*_,*V*_*acf*_), are normalized because of the uneven scale. DBSCAN clustering method was used to cluster according to feature vectors, which classified IMFs with similar stationary characteristics into same group. ED is used to solve the distance of all vectors, then the minimum distance value of the calculated vector is found out, and the group with the minimum value was marked as "ML-group", and the other group is marked as "DL-group". The calculation formula of distance is as follow.

$$dist(IMF)=\sqrt{{V_{FE}}^{2}+{V_{acf}}^{2}}$$ (8)

where *V*_*FE*_ represents FE value and *V*_*acf*_ represents the autocorrelation coefficient.

Subsequently, all of the IMFs were dichotomized by the clustering algorithm, and the Euclidean distance was used to solve the distance of all vectors, then the minimum distance value of the calculated vector was found out, and the group with the minimum value was marked as "ML-group", and the other group was marked as "DL-group".

### Prediction model

In this paper, we use a combination of machine learning and deep learning to predict data. The machine learning model used in the paper is adopted as OSELM, which is currently a popular prediction model due to its ability to effectively capture the inline relationships of nonlinear and non-stationary data. In another way, this machine models developed from extreme learning machines can process feed-in input data sets continuously. For better performance of prediction, this machine learning model is optimized by training dataset. In the ED-CNN-BiGRU-Att model as machine learning in this paper, of which layers include convolutional neural network (CNN) layer, pooling layer, bidirectional gate recurrent unit (BiGRU) layer, and self-attention layer, and its overall organizational structure uses encoder-decoder mode for data interaction. In the overall structure, the CNN is used to obtain the one-dimensional data characteristics of oil production. The BiGRU is used to learn the characteristics of oil production time series and strengthen the information of the front data. The self-attention layer retains the probability weight of the hidden state of the time characteristics of the oil production series in the learning process, and redistributes the characteristic parameters of the key information of the data. The Encoder-Decoder framework shares the learning weight. The Encoder pre-trains the dataset, extracts the data information, and forms low-rank vector. Then the Decoder processes and extracts the information and finally outputs it, so as to retain the effective information of the data to the greatest extent.

#### Online sequential extreme learning machine model based on whale optimization algorithm

Whale optimization algorithm (WOA), as the algorithm to optimize the hyper parameters of ELM model in this study, was first proposed by Mirjalili in 2016 imitating the unique predatory behavior of humpback whale population [32], where the processes included helical local search and random search. WOA imitated the hunting process of hump back whales, that is, prey enclosure, bubble attack, searching for prey, to search for the optimal, and adopted helical local search and random search to avoid falling into local optima.

As the ML of sub model in this study, online sequential extreme learning machine (OSELM), improved from ELM, is a single hidden layer feed-forward network [33]. Inheriting the advantages of ELM, such as simple structure, fast training speed, and strong generalization ability, OSELM can continuously update the model with the new arrival data, instead of retraining. However, OSELM needs to determine its main hyper parameters when initializing the training. Using the empirical method to set parameters is a common means. Still, the empirical value often has a certain subjectivity, which makes OSELM unable to accurately predict the trend and direction of data. In addition, the OSELM model only contains a small number of hidden layers, and the weight parameter volume of model training is small. The model training process has fewer parameters, less time consumption and low trial and error cost. Moreover, the whale optimization algorithm does not need complex calculations and can achieve faster search speed through a small number of parameters. At the same time, it can avoid falling into local optimization and help OSELM quickly search for the optimal parameter value.

The number of units *n* and pseudo random number generator *R* as two important parameters of OSELM are optimized by WOA, in which MSE of the training data and predicted value by OSELM is given as loss function. Finally, WOSELM obtained through the above process is used as the machine learning model in this paper.

### Deep learning network

In this study, CNN is chosen as the protrusion layer because of its ability to map the data to multiple different scales to extract data features through convolutional operations [34]. However, it is difficult to identify the time series causality of the internal data of single well production time series data. In order to obtain the timeseries association information of the data set, the GRU network layer is added to analyze the time series data association information, and the information is extended backward and retained. GRU is a variant of the recurrent neural network after streamlining the internal structure of LSTM, whose internal structure includes a reset gate and an update gate composed of different activation functions, as shown in S3 Fig. The specific mathematical expression of interna data flow of GRU is as follows:

$$\{\begin{array}{c} z_{t}=\sigma (W_{z}\cdot [H_{t-1},x_{t}])\\ r_{t}=\sigma (W_{r}\cdot [H_{t-1},x_{t}])\\ {\tilde{H}}_{t}=\tanh(W\cdot [r_{t}*H_{t-1},x_{t}])\\ H_{t}=(1-z_{t})*H_{t-1}+z_{t}*{\tilde{H}}_{t} \end{array}$$ (9)

where *W*_*z*_, *W*_*r*_ and *W* are weight coefficients; *H*_*t*-1_ is the implicit information, and *H*_*t*_ represents the intermediate hidden content.

Although the GRU can extract the correlation characteristics of data time scale, it will continue to transfer back calculation with the amount of time series, weakening or even losing the previous correlation information.

Therefore, this paper uses bidirectional Gated Recurrent Unit (BiGRU) to construct deep learning network instead of GRU, which can relearn the features of the previous data [35], as shown in S4 Fig. The network stacks a one-dimensional CNN layer, max-pooling layer, BiGRU and linear layer. Through the unique recursive reverse link mode of BiGRU, it completes forward propagation and then backpropagation, to retrieve the front-end time series data information, ensure the continuity of time series information, and strengthen the front-end time series information. In order to enable CNN-BiGRU to consider the global information fully, the attention mechanism is introduced to allocate the calculation weight of the sequence value to obtain the global information of the data [36]. The output of the CNN-BiGRU network is taken as the three components of *Q*, *K* and *V*, which are transformed by the linear laver and then calculated and output according to the dot product of the following formula.

$$Attention\left(Q,K,V\right)=softmax(\frac{QK^{T}}{\sqrt{d_{k}}})\cdot V$$ (10)

where *K*^*T*^ represents the transpose matrix of the last two dimensions of the *K* component, *d*_*k*_ represents the dimensions of *Q* and *K*, $\sqrt{d_{k}}$ is regarded as a scaling factor, and *softmax* is a normalized exponential function, which can obtain the weight information vector containing the probability of primary and secondary characteristics of the data. The introduction of attention mechanism calculation can enhance the main characteristics of output data and weaken secondary information.

To obtain the sequence feature information in advance and ensure the learning efficiency of the deep network model for data, the above network is optimized and constructed under the encoder-decoder framework to form the ED-CNN-BiGRU-Att network model, as shown in S4 Fig. The network encoder comprises a convolution layer, a pooling layer and a bidirectional gate recurrent unit network layer. The input data is trained and learned in the decoder, and the hidden weight information is output by the last layer. At the same time, after the input data passes through the one-dimensional convolution layer and the max- pooling layer in turn, it combines the implicit weight information returned by the encoder to construct two inputs of the subsequent two-way cyclic network layer. It continues to pass forward into the self-attention layer, and finally the linear layer is the output of the overall network structure.

### Data fusion model

After the construction of ML and DL, IMFs are predicted by the two models respectively after redistribution to play their respective advantages.

The series both in “ML-group” and “DL-group”, generated through the step of data distribution process above, are separately added together to build new two series “ML-series” and “DL-series”. Then as illustrated in S5 Fig, “ML-series” and “DL-series”are predicted by WOSELM and ED-CNN-BiGRU-Att, respectively, after which both of the predicted are summed to obtain the final result.

## Data sources

In this paper, the dataset is selected from the historical data of a well in the Gudao oil production plant in the Shengli oilfield since 2005, as shown in S6 Fig. The well is located in a heavy oil reservoir currently in a high water cut phase with a cumulative production of 17,333.6 tons. Three oil well production datasets with different production periods, scales and characteristics are selected, and time phases are May 30, 2007 to March 19,2009 (early period), May 30, 2009 to April 28, 2011 (mid period) and 2018 to July 8,2020 (late period).

The statistical analysis of the selected three-section production dataset is shown in S1 Table. It can be seen that the average oil production of dataset 1 in the early stage of production is high, and the oil production is affected by the initial geological state of the oil-producing formation and equipment adjustment, showing a large fluctuation. For dataset 2 in the mid period, the fluctuation characteristics are weakened, because the production equipment parameters and production scheme are more stable, while the local adjustment of production parameters will also bring about large fluctuations in oilwell production. Data set 3 in the late stage of production due to the change of the connectivity between oil and water wells compared with before. Affected by the increase of water injection for water drive oil production and other auxiliary production methods, the water content of oil production is higher, the corresponding average oil production is lower and more stable, and the fluctuation range is very small.

Besides, all the code involved in this study is used python 3.8.2 and the deep learning framework is based on pytorch 1.10.1. The computer hardware possesses the CPU of Intel®) i7-11700K @3.6GHz and GPU of GeForce®) GTX1660Ti with 8 GB ROM.

## Results and discussion

### Data preprocessing and analysis

After decomposition by CEEMDAN, seven IMFs and one residual are obtained, as shown in S7 Fig. The residual component cannot continue to be decompose, which is discarded in this study. IMFs1-IMFs3 are high-frequency signals, IMFs4 and IMFs5 are medium-frequency signals, and IMFs6 and IMFs7 are low-frequency signals that reflect the data trends of the actual values.

Entropy is a measure that can describe the complexity and chaos of a time series signal. The larger its value, the more complex the waves of the sequence signal, and the more frequencies it contains accordingly [31]. Moreover, AC can also express correlations before and after data points in a time series dataset to characterize data complexity. FE and AC of IMFs after normalizing are given in S2 Table. It is clear seen in S8 Fig that decomposition can significantly reduce the entropy value of the raw data, resulting in a set of IMFs with reduced complexity. This process can further extract the influence factors of oil production from the data, which is beneficial for subsequent model training and learning. In S2 Table, the label of DL represents the “DL group” after clustering and grouping, while ML represents the “ML group”.

### Comparison analysis of prediction performance

Taking dataset 1 as an example, it is divided into a training set and test set according to the ratio of 8:2, and processed according to the short-term prediction method of single well production based on the multi-agent hybrid model proposed in this paper. First, CEEMDAN is used to decompose and calculate the training set data, and seven groups of IMFs and a residual quantity can be obtained as shown in S7 Fig. Among them, the residual quantity is the smallest component of the order that cannot be decomposed after decomposition and reconstruction, which can be considered as the noise quantity after decomposition and can be ignored. It can be seen from S7 Fig that the original data is decomposed into components with less frequency, and the frequency range covers high frequency, intermediate frequency and low frequency. IMFsl-IMFs3 are high-frequency signals, and the data floating range is within the range of [-2.5, 2.5]. The magnitude is smaller than the original data, which can be regarded as random signals; IMFs4 and IMFs5 are intermediate frequency signals. The data floating range is the same as that of high-frequency signals, while their frequency is lower than that of high-frequency signals. They show a certain periodic law, which can be regarded as quasi-periodic signals generated after decomposition; IMFs6 and IMFs7 are low-frequency signals that reflect the actual value’s data trend and can be considered as the trend quantity information. Among them, the amplitude range of IMFs6 is the same as that of high and intermediate frequency, showing a concave curve similar to the data form of the actual value. IMFs7 is the component with the lowest frequency, and the amplitude is the closest to the actual value, showing a gradual decreasing trend, which is consistent with the gradual decreasing trend of the actual value. The above calculation can decompose the data training set into IMFs with different frequencies, and various features implied in the actual value data can be characterized in a data-driven manner.

Since the decomposed and reconstructed IMFs component is one-dimensional data, the sliding window method converts the original one-dimensional data into a data form to generate a data set that the model can identify. Each set of data in the dataset contains 10 predictive training values and 1 tag value. At the same time, to avoid identifying the time correlation characteristics and improve the generalization ability of model prediction, the dataset is randomly mixed.

For the IMFs components with different characteristics in dataset 1, the FE value and autocorrelation coefficient are used to construct the sequence feature vector, which is used as the classification basis for clustering. Due to the different scales of FE value and autocorrelation coefficient, the maximum and minimum normalization method is used to normalize FE value and autocorrelation coefficient, and the results are shown in S2 Table.

Since entropy is a measure of the complexity and chaos of sequence data, the greater its value indicates that, the more frequent the data sequence contains, the greater the chaos of the sequence [30], which can accurately reflect the complexity of daily oil production data of a single well. Therefore, this paper uses the Fuzzy Entropy value and autocorrelation coefficient to quantify the complexity of the data decomposition and reorganization sequence, and constructs the feature vector based on this, combined with the data clustering theory, as the basis for sub-model data grouping input.

The IMFs components were clustered using density-based clustering, and the number of centers was set to 2. The IMFs components were divided into two groups, IMFs1-IMFs4 and IMFs5-IMFs7. Calculating the euclidean distance of the sequence eigenvector, the group of the minimum length is obtained. This data group has low The IMFs components were clustered using density-based clustering, and the number of centers was set to 2. The IMFs components were divided into two groups,IMFs1-IMFs4 and IMFs5-IMFs7. Calculating the euclidean distance of the sequence eigenvector, the group of the minimum length is obtained. This data group has low non-stationary but strong volatility, complex data components, and high pre and post-dependence. Therefore, this group uses the deep learning model for training prediction, while the other group uses the machine learning model. The FE value, autocorrelation coefficient, and grouping obtained through the above clustering process are shown in S2 Table. According to the above clustering grouping, the accumulated values of IMFs1-IMFs4 and IMFs5-IMFs7 components are predicted respectively by ED-CNN-BiGRU-Att deep learning model and OSELM machine learning model based on the WOA algorithm, and then the two groups of predicted values are fused and accumulated to finally obtain the single well production prediction value.

According to the short-term prediction method of oil field single well production based on the multi-agent hybrid model, the selected three-stage data set is predicted. The results of IMFs component FE value obtained from the training set data of the three-segment data set and its decomposition are shown in S8 Fig. It can be seen that the mean value of data set 1 is high, the fluctuation is large, and the FE value of data is large, although the FE value of IMFs component is significantly reduced after decomposition: The FE values of the mean, volatility and decomposition of dataset 2 are lower than those of dataset l; Dataset 3 has the smallest amplitude change, the best stability, and the FE value of the original data and IMFs component is the lowest. Therefore, CEEMDAN decomposition can effectively reduce the complexity of the dataset, and can be quantified by FE value. The FE values of the selected three data sets and their IMFs components all show a downward trend one by one. It can be seen that the FE value of the IMFs component of the single well daily oil production time series data is less than the original data, indicating that the data decomposition and reconstruction method can effectively reduce the degree of data confusion and non-stationarity and can separate the influencing factors of oil production from the, which is conducive to the prediction of subsequent models.

The training sets of the three datasets are further divided into training and validation sets, which are used for tuning hyperparameters of the machine learning and deep learning models. Due to the incorporation of key components such as OSELM, GRU, and linear layers in the proposed predictive model of this study, comparative models including OSELM, BP, LSTM, and CNNBiGRU are included in the predictive research.

Based on the characteristics of oil production data, the main hyper parameters of the ED-CNN-BiGRU-Att network model are given as follows: The out channels value is 32 and the kernel size is 2 in the CNN, respectively. The kernel size is 2 in the pooling layer. In the BiGRU, the hidden size is set to 12, the num layers is set to 3, and bidirectional is set to true value, The number of neurons in the linear layer in the Attention is also set to 12. Also, LSTM model contains 12 intermediate cell layers, amd the model is trained for 2000 iterations with a learning rate of 2E-4. S9 Fig shows the predictive performance of the various models across the three datasets, displayed by the predicted line plots and the predicted Taylor plots. From the prediction line plots, it can be observed that the predicted values of all models are closely aligned with the actual values, indicating that each model exhibits excellent predictive power for well production. Across the three datasets, all models exhibit some level of hysteresis, but the proposed model in this study demonstrates the best tracking performance in terms of its predicted values.

The Taylor diagram is a widely used and provides a comprehensive assessment of multiple model variables by comparing their statistical properties with observations. The diagram consists of a scatterplot that displays the standard deviation and correlation coefficient of each model variable relative to the reference dataset. The distance between each model point and the reference point represents the standard deviation ratio, indicating the spread of the model variable compared to the observed data. A smaller distance suggests a better agreement between the model and observations in terms of variability. From the Taylor diagram, it is evident that the points of the proposed model are noticeably closer to the other models compared to the other models. Secondly, the angle between the model variable vector and the reference vector represents the correlation coefficient. A small angle indicates a strong correlation between the model and observed data, implying a good match in terms of pattern and phase. From this perspective, the proposed model in this study is also more competitive.

To quantify the short-term prediction effect, performance metrics are selected, including Mean Absolute Error (MAE), Mean Absolute Percentage Error (MAPE), Root Mean Squared Error (RMSE), R-Square (R^2^), Mean Absolute Range Normalized Error (MARNE) [37], and Nash-Sutcliff efficiency (NSE) [38], of which the formulas are as follows:

$$MAE=\frac{1}{N}\sum_{i=1}^{N}\left|{\hat{y}}_{i}-y_{i}\right|$$ (11)

$$RMSE=\sqrt{\frac{1}{N}\sum_{i=1}^{N}{\left({\hat{y}}_{i}-y_{i}\right)}^{2}}$$ (12)

$$MAPE=\frac{1}{N}\sum_{i=1}^{N}\left|\frac{{\hat{y}}_{i}-y_{i}}{y_{i}}\right|\times 100\%$$ (13)

$$R^{2}=1-\frac{\sum_{i=1}^{N}{\left({\hat{y}}_{i}-y_{i}\right)}^{2}}{\sum_{i=1}^{N}{y_{i}}^{2}}$$ (14)

$$MARNE=\frac{1}{N}\sum_{i=1}^{N}\frac{\left|{\hat{y}}_{i}-y_{i}\right|}{max({\hat{y}}_{i})}\times 100\%$$ (15)

$$NSE=1-\frac{\sum_{i=1}^{N}{\left({\hat{y}}_{i}-y_{i}\right)}^{2}}{\sum_{i=1}^{N}{\left(\bar{y}-y_{i}\right)}^{2}}$$ (16)

where, ${\hat{y}}_{i}$ is the predicted value calculated by the prediction model, *y*_*i*_ is the original data value in the test set, and $\bar{y}$ is the predicted mean value.

In addition, the Diebold-Mariano (DM) test method is a test method to evaluate the prediction performance from a statistical perspective [39, 40]. Eq (17) gives the hypothesis that the prediction performance of the model in this paper is better than that of the comparative model.

$$\left\{ \begin{array}{l} H_{0:}:E(m_{t}^{d}-m_{t}^{c})=0\\ H_{1:}:E(m_{t}^{d}-m_{t}^{c})\neq 0 \end{array}\right.$$ (17)

where *H*_0_ and *H*_1_ represent the original hypothesis and alternative hypothesis respectively. $m_{t}^{d}$ and $m_{t}^{c}$ represent the mean square error between the predicted value and the real value of the model, respectively.

In statistical methodology, the Kruskal-Wallis test (KW test) does not require the assumption of a normal distribution for the data under investigation. Therefore, for the prediction of oil production data, the KW test is employed to evaluate the differences in the predicted values between the different models in this paper. The calculation for KW test is as follows:

$$KW=\frac{12}{n(n+1)}\sum_{i=1}^{k}n_{i}{\left({\bar{R}}_{i}-\bar{R}\right)}^{2}$$ (18)

In the equation above, *k* is the number of control factor layers, *N*_*i*_ is the number of samples generated by the *i*th hierarchy, ${\bar{R}}_{i}$ is the average rank generated by the *i*th hierarchy, and $\bar{R}$ represents the average rank generated by *k* levels, whose value is equal to (*n*+1)/2.

According to the above prediction effect evaluation method, the results of the statistical value of the predicted data deviation of the three-segment dataset model are shown in S3 Table, while DM test and KW test values are given in S4 Table. According to MAE, MAPE, RMSE, R^2^, MARNE and NSE values, the prediction performance of this method has obvious advantages over other models. Taking LSTM as an example for comparison, the MAE value predicted by this model for three data sets is 49.94%, 57.94%, and 16.24% lower than that of the LSTM model, respectively. For the MAPE index, the MAPE value predicted by this model for the three data sets is 52.03%, 58.07%, and 18.93% lower than that of the LSTM model, respectively. For the RMSE index, the RMSE value predicted by this model for the three data sets is 52.53%, 66.89%, and 44.21% lower than that of the LSTM model, respectively. For the R^2^ index, the predicted R^2^ value of this model for the three data sets is 22.90%, 20.01%, and 41.59% higher than that of the LSTM model, respectively. For the MARNE index, the MARNE value predicted by this model for the three data sets is 50.52%, 57.63%, and 16.09% lower than that of the LSTM model, respectively.

In addition, the p-value of the DM test of the proposed model in S4 Table is less than 0.05 on the three data sets, so the original hypothesis can be considered not to be tenable, and because the DM values are negative, it can be considered that the prediction effect of this model is better than that of LSTM model. The difference of the mean sample ranks between the level using the comparative method and proposed method is exhibit in S4 Table via KW test as well. It is indicated that the proposed model does not exhibit significant differences compared to other models in terms of minimizing the deviations from the test values form the KW test results in Dataset 1, which may be attributed to the presence of substantial fluctuations and does not necessarily imply a disadvantage of the proposed model. Nevertheless, in the other datasets, the data behaves differently, which demonstrates that the proposed model has minimal differences between it and the true values compared to the others.

In left side of S10 Fig, the violin plots of raw data with five model predictions are presented to compare their predictions, considering an intuitive statistical representation for the prediction results of models [11]. It can be observed that across the three datasets, the predicted values of each model exhibit remarkable similarity in terms of upper and lower quartiles, median values, and overall graph shape, which indicates that the predicted values of the models are generally aligned with the actual values. From an intuitive statistical point of view, the predicted value of the proposed model is closer to the test value. In right side of S10 Fig, The error boxplots for each model depict the deviation of the predicted values from the actual values across the three datasets. The proposed model overlooks the noisy components in the data, resulting in a smaller overall error and fewer outliers in the boxplots compared to the other models.

Fit plots are commonly used to analyze the predictive results. The distribution of predicted value scatter plots and the angle between the scatter fit line and the actual value centerline are used to determine the prediction performance. In S11 Fig, it is evident that the predicted values of the proposed model are more concentrated compared to the scatter plots of the other compared models across the three datasets. Moreover, the fitted line of the proposed model has the smallest angle with the center line of the actual value.

## Conclusion

Accurate and effective short-term prediction of single well production can provide direct data support for timely optimization and adjustment of key parameters in oilfield production and research and judgment of oil well production conditions. This paper proposes a short-term prediction method of single well production based on a multi-agent hybrid model. The typical data of oil wells in different periods are selected for prediction and analysis, and the prediction effect is evaluated. The following conclusions are obtained:

1. Considering that the short-term time series data of daily oil production of a single well are affected by many factors, such as porosity, permeability, oil-water saturation, oil-water viscosity ratio of crude oil and inter-well connectivity, showing greater randomness and volatility, and the internal mechanism of the data is relatively complex, this paper proposes a short-term prediction method for oil production of a single well in the oilfield by combining data decomposition and multi-agent combination model. The short-term prediction process of oil production per well in the oilfield is constructed. The WOSELM machine learning model based on the whale optimization algorithm and the convolutional bidirectional gated cyclic network model combined with self-attention in the Encoder-Decoder framework is proposed as the prediction sub-model. The prediction method is constructed through data decomposition, data set generation, component aggregation, model allocation, and prediction fusion. By using this prediction method to predict and analyze the typical data of oil wells in different periods, the effectiveness of this prediction method in predicting the short-term production of single well daily oil production is verified.
2. According to the daily production data of a single well with high fluctuation and poor stability, the CEEMDAN method decomposes the data. Clustering and grouping fusion are used to effectively reduce the data’s complexity through evaluating and analyzing the FE value of IMFs components obtained after decomposition. This decomposition method can decompose the data training set into IMFs components with different frequencies, which can characterize the various characteristics implied in the actual value data in a data-driven way. Separating the influencing factors of oil production can effectively weaken the degree of data chaos and non-stationary, which is conducive to predicting subsequent models.
3. The statistical value of data deviation and statistical test methods is introduced as the basis for comparison and evaluation. The short-term prediction effect of the prediction model proposed in this paper and the contrast models commonly used in data prediction on single well daily production in different periods is compared. Taking R^2^ as an example, the predictive performance metrics of the proposed model across the three datasets are 0.997, 0.998, and 0.997, all of which exceed the metrics of other models. The results show that according to the comparison of the statistical value of data deviation, this model has better prediction generalization ability and higher prediction accuracy than the contrast models. The statistical test methods introduced in this paper can verify that the prediction effect of this model is better than that of the contrast models.

Although the excellent predictive performance of the model presented in this study has been thoroughly validated on real data, it still has certain limitations. First, factors related to oil production, which often constrain or affect the outcome of oil production, were not considered. Exploring these oil production factors should therefore be a direction for future research. Second, researchers should continue to optimize the model to reduce its time complexity. Finally, in terms of methodology, it is recommended that the proposed model will be continuously improved to adapt to different oilfield application scenarios and further improve the accuracy of model prediction.

## Supporting information

S1 Fig Detailed steps of the proposed method. (TIF)

S2 Fig Flow chart of sliding window. (TIF)

S3 Fig Internal structure of GRU. (TIF)

S4 Fig Structure of convolutional neural network and bidirectional gating neural network combined with self attention mechanism under encoder decoder framework. (TIF)

S5 Fig Structure of convolutional neural network and bidirectional gating neural network combined with self attention mechanism under encoder decoder framework. (TIF)

S6 Fig Historical data of oil well production. (TIF)

S7 Fig Original data of training set in dataset 1 and IMFs component curve after decomposition by CEEMDAN. (TIF)

S8 Fig FE value of three segment data training set, original data and IMFs component. (TIF)

S9 Fig Predicted value curve and Taylor diagram of this model and the comparison model on the three-stage three datatsets. (TIF)

S10 Fig Violin plots of predicted values for each model across three datasets. (TIF)

S11 Fig Fit plots of predicted values for each model across three datasets. (TIF)

S1 Table Statistics of characteristics of oil well production data set. (TIF)

S2 Table Normalized values of fuzzy entropy and autocorrelation coefficient and clustering results of dataset. (TIF)

S3 Table Predicted performance index values of three dataset model. (TIF)

S4 Table Statistical tests values of three dataset model. (TIF)
