# Supplementary figures and images for: Short-term forecasting approach of single well production based on multi-intelligent agent hybrid model
Source: PLoS One. 2024 Apr 17;19(4):e0301349. doi: 10.1371/journal.pone.0301349 (PMC11023203; doi:10.1371/journal.pone.0301349)

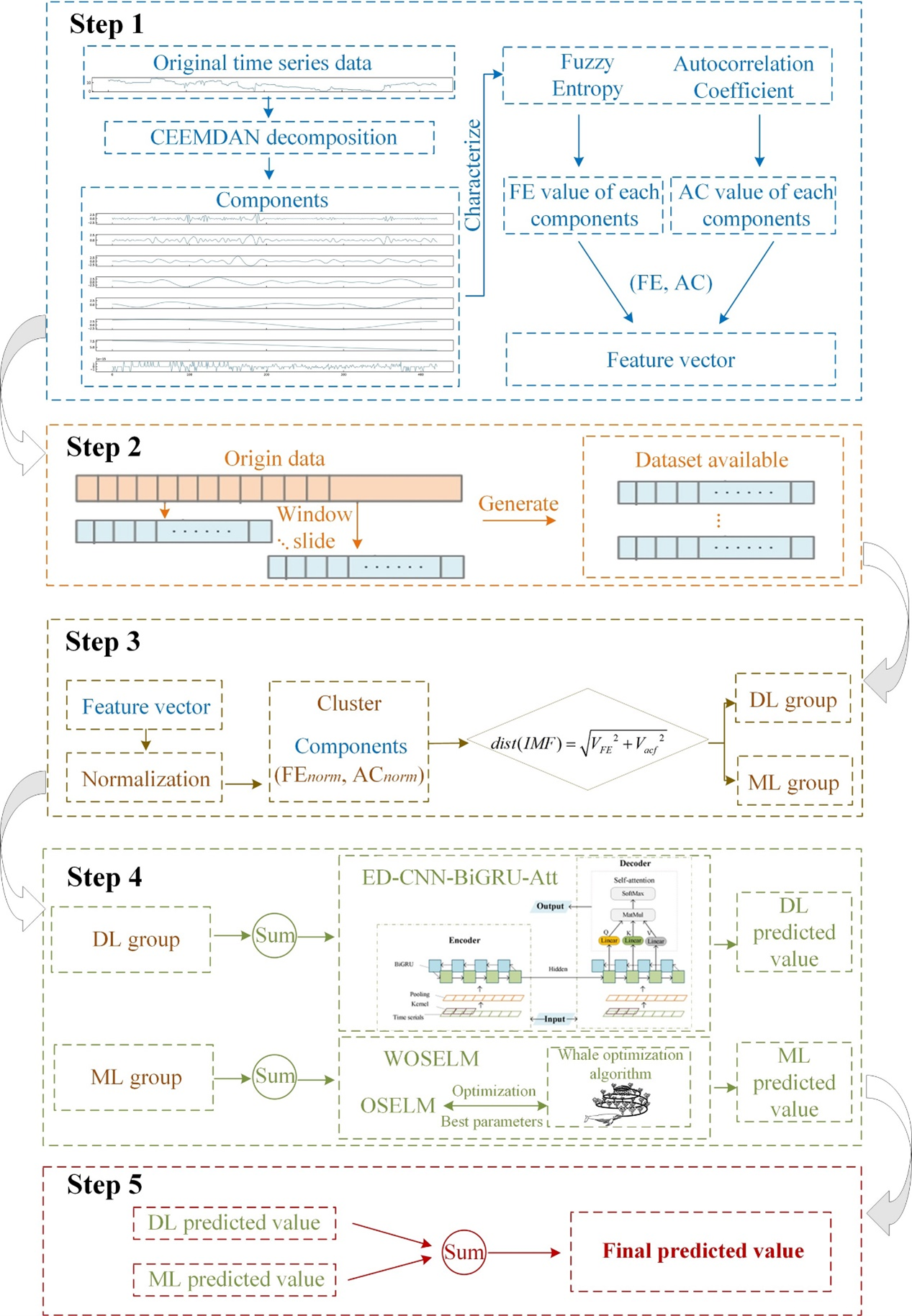

Supplement: S1 Fig — (TIF) [file pone.0301349.s001.tif]

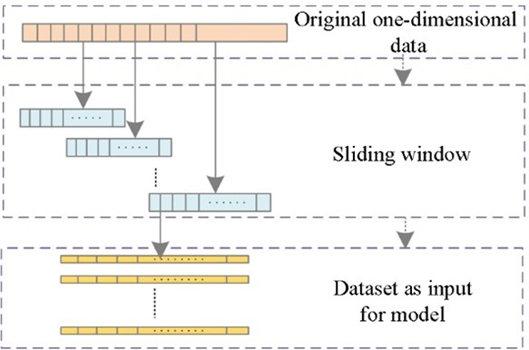

Supplement: S2 Fig — (TIF) [file pone.0301349.s002.tif]

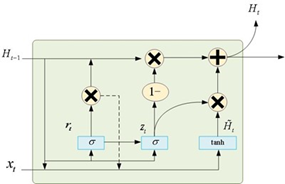

Supplement: S3 Fig — (TIF) [file pone.0301349.s003.tif]

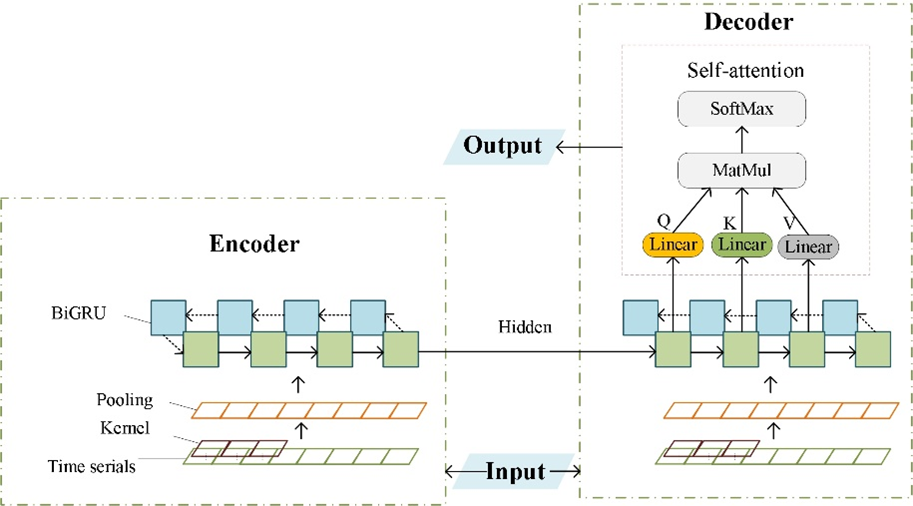

Supplement: S4 Fig — (TIF) [file pone.0301349.s004.tif]

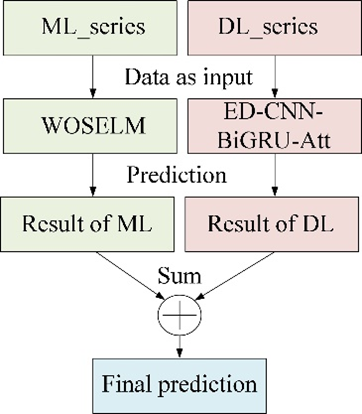

Supplement: S5 Fig — (TIF) [file pone.0301349.s005.tif]

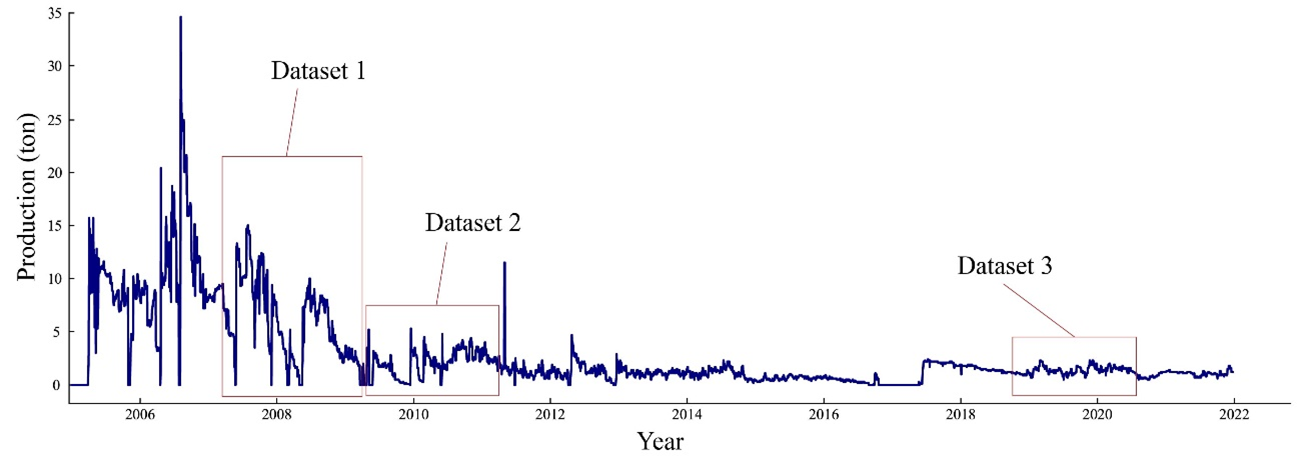

Supplement: S6 Fig — (TIF) [file pone.0301349.s006.tif]

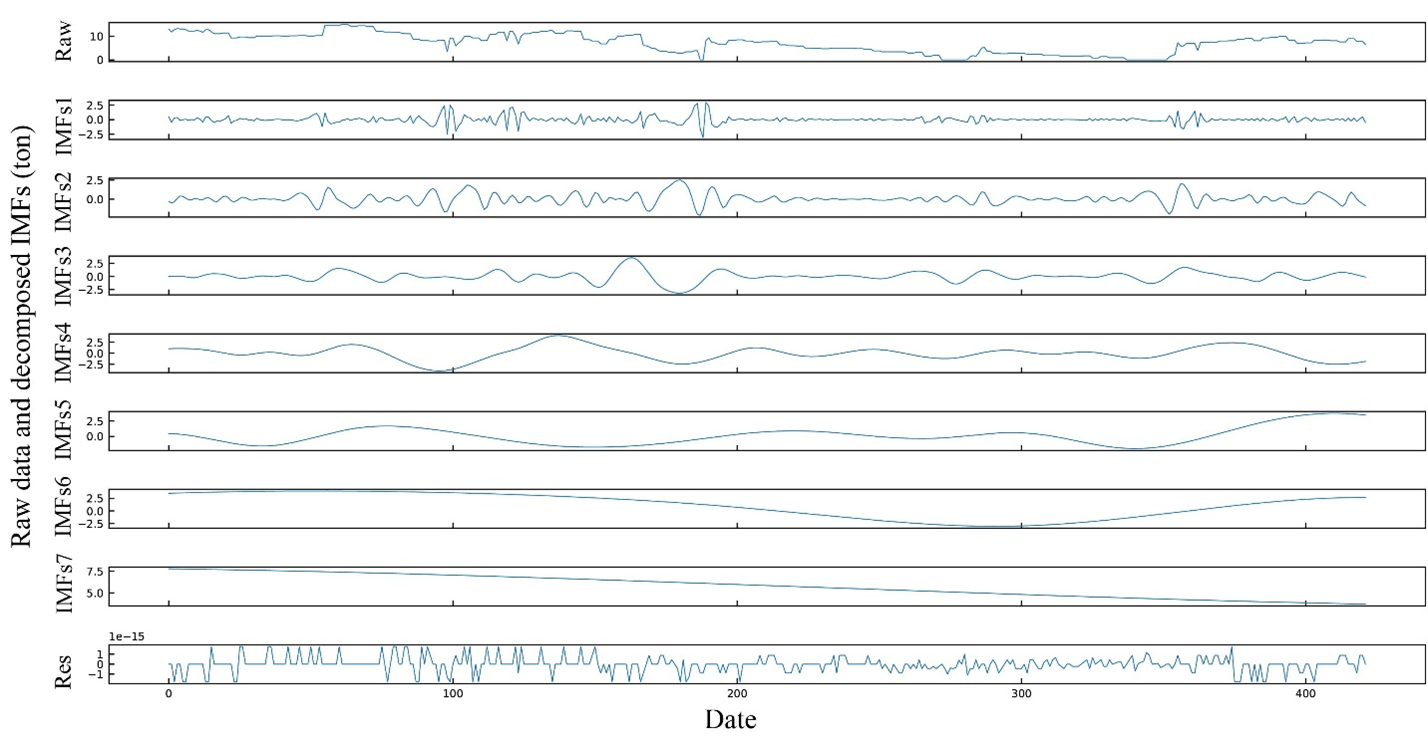

Supplement: S7 Fig — (TIF) [file pone.0301349.s007.tif]

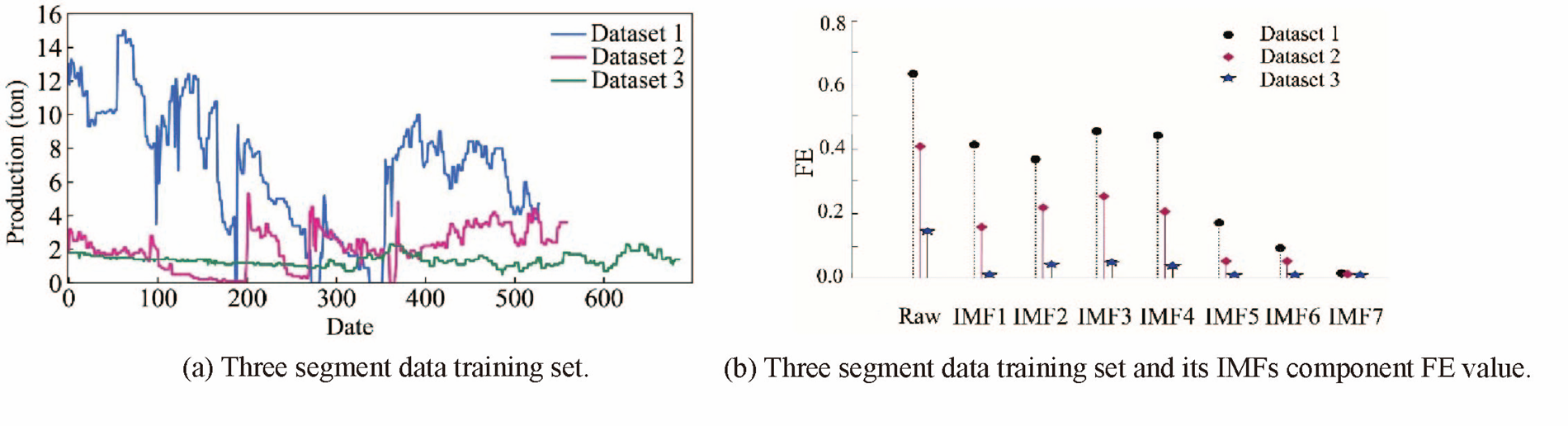

Supplement: S8 Fig — (TIF) [file pone.0301349.s008.tif]

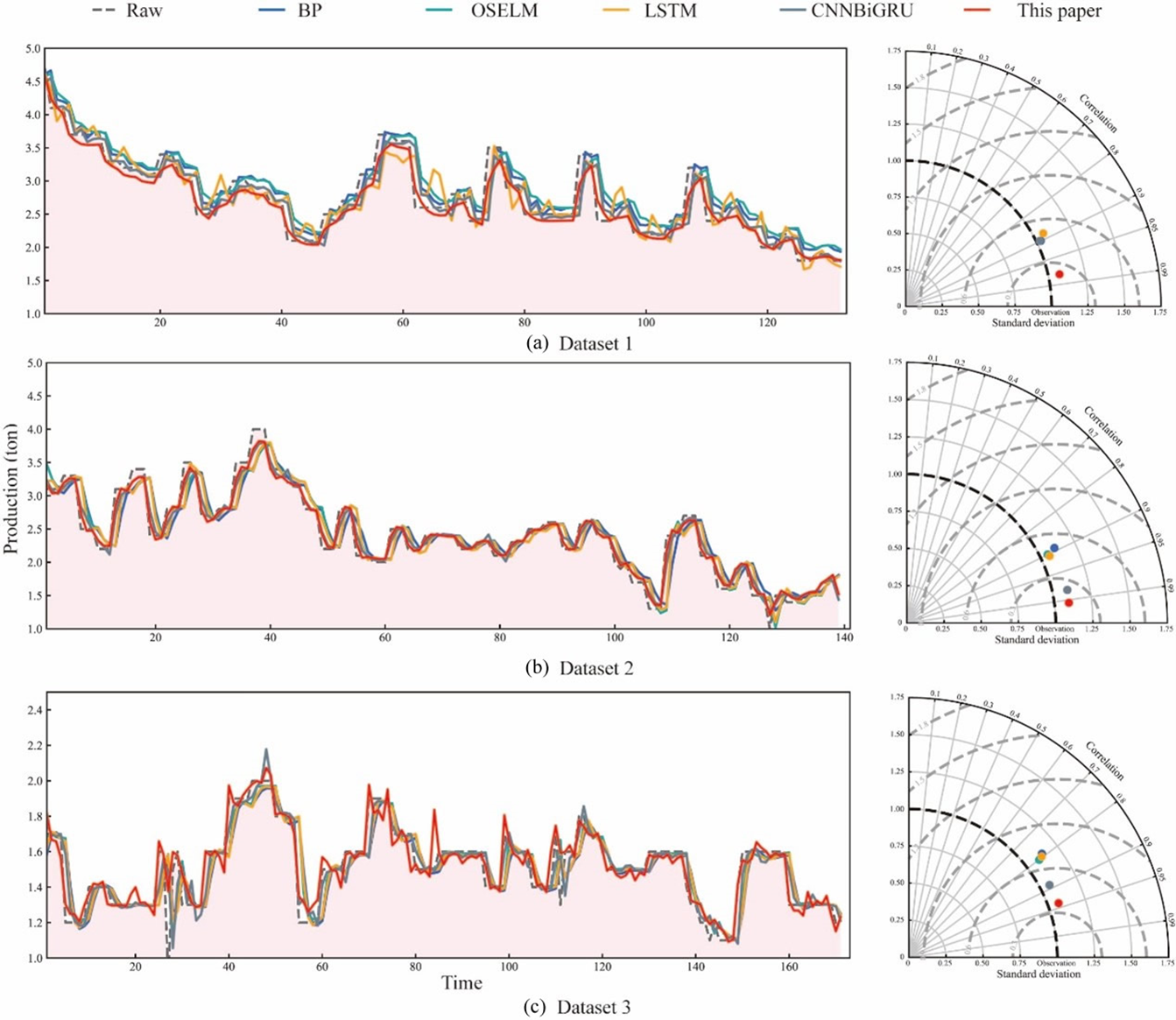

Supplement: S9 Fig — (TIF) [file pone.0301349.s009.tif]

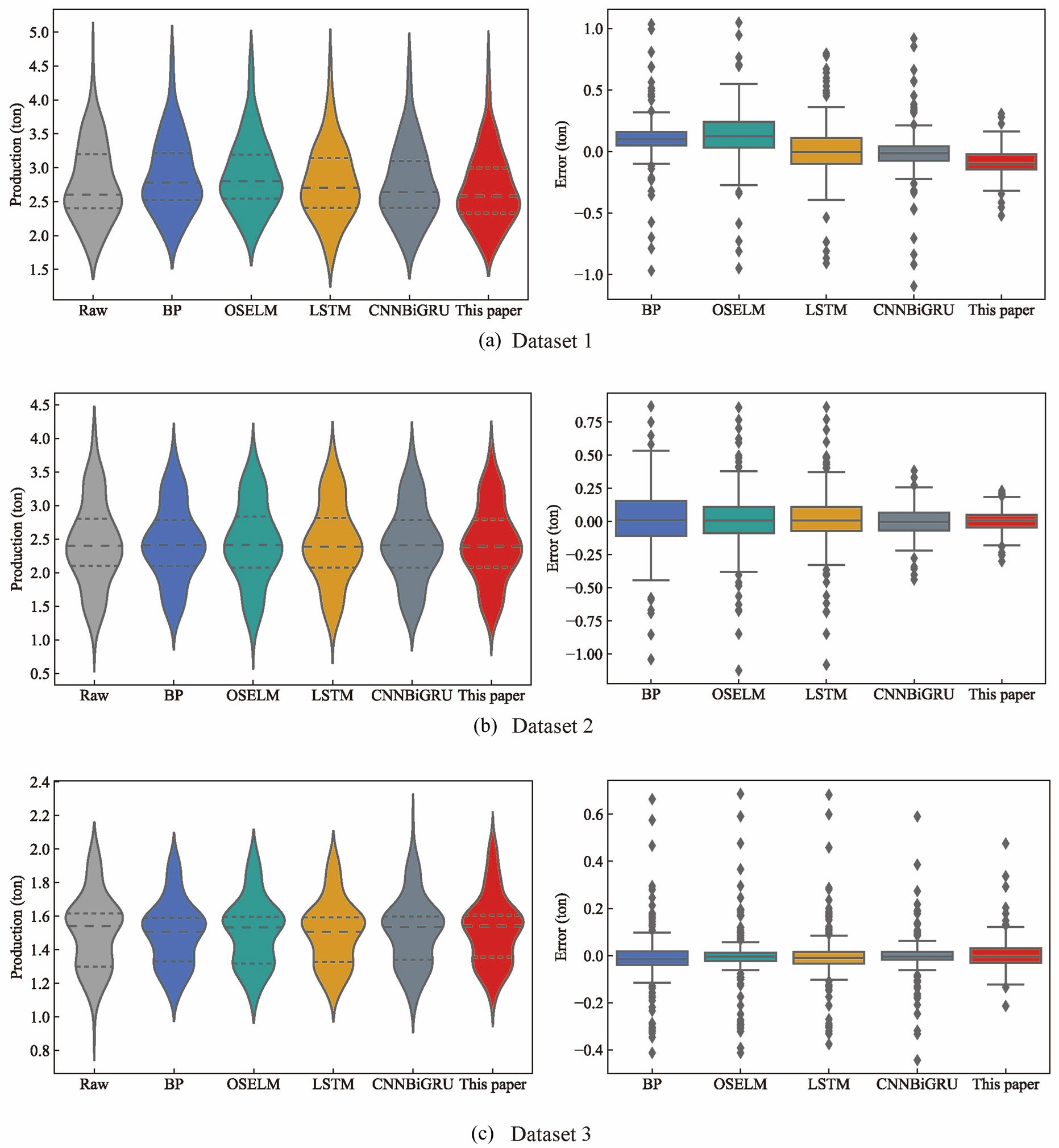

Supplement: S10 Fig — (TIF) [file pone.0301349.s010.tif]

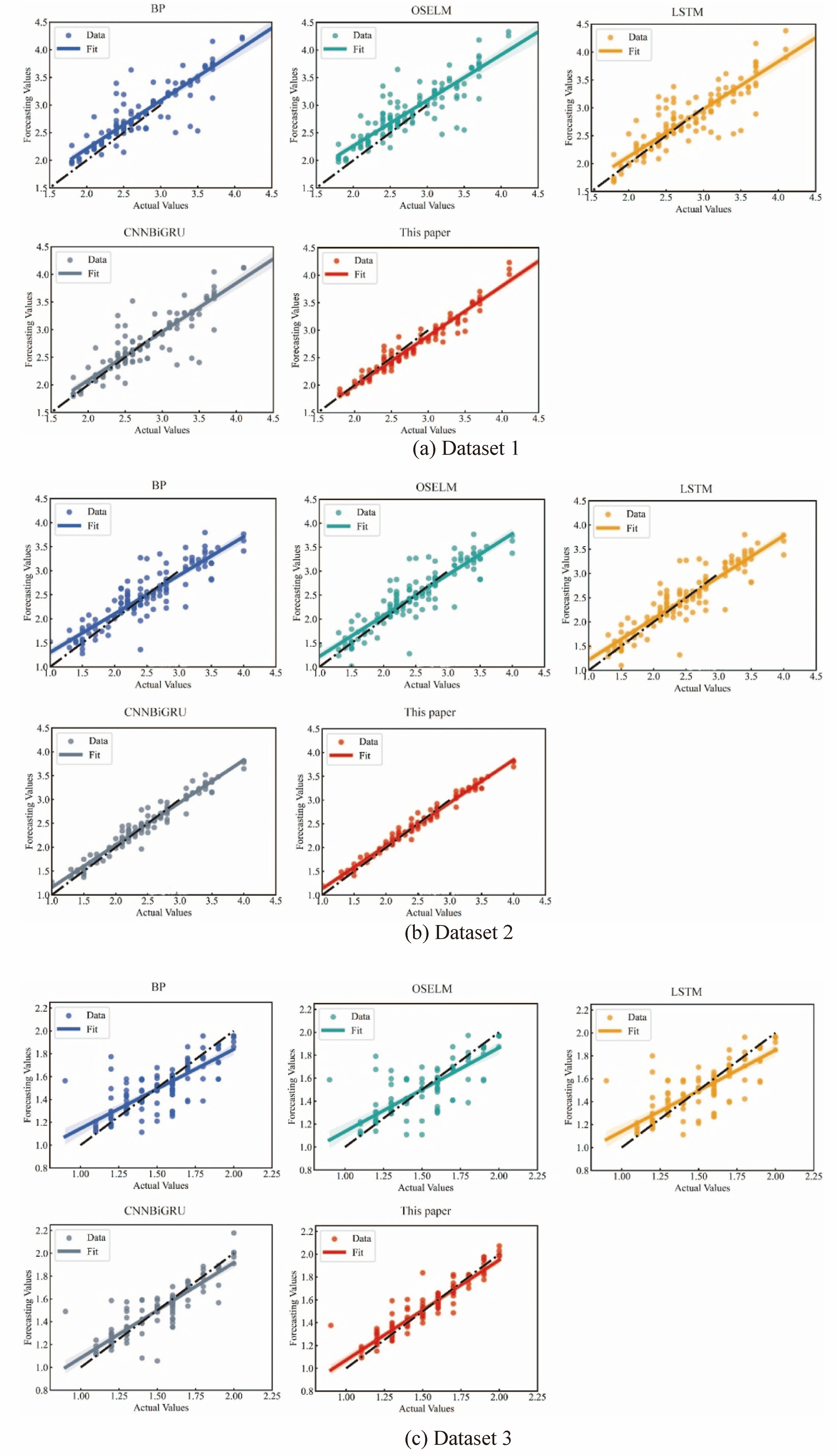

Supplement: S11 Fig — (TIF) [file pone.0301349.s011.tif]

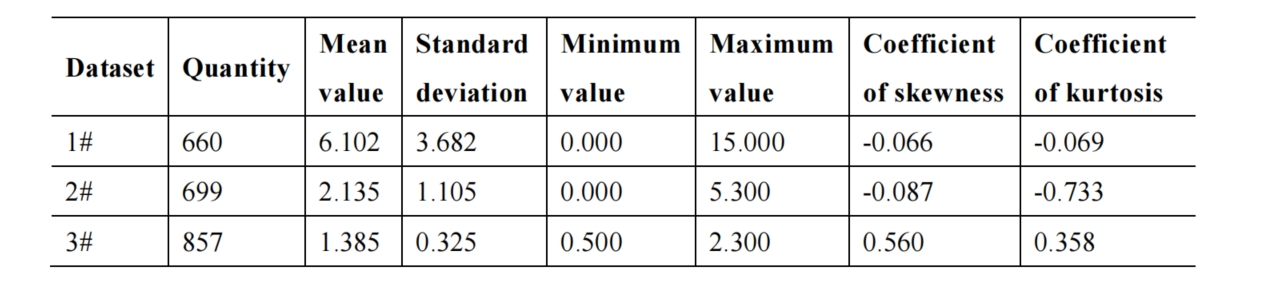

Supplement: S1 Table — (TIF) [file pone.0301349.s012.tif]

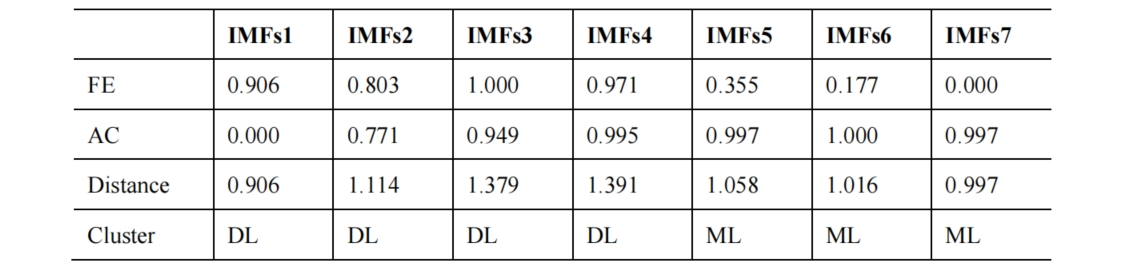

Supplement: S2 Table — (TIF) [file pone.0301349.s013.tif]

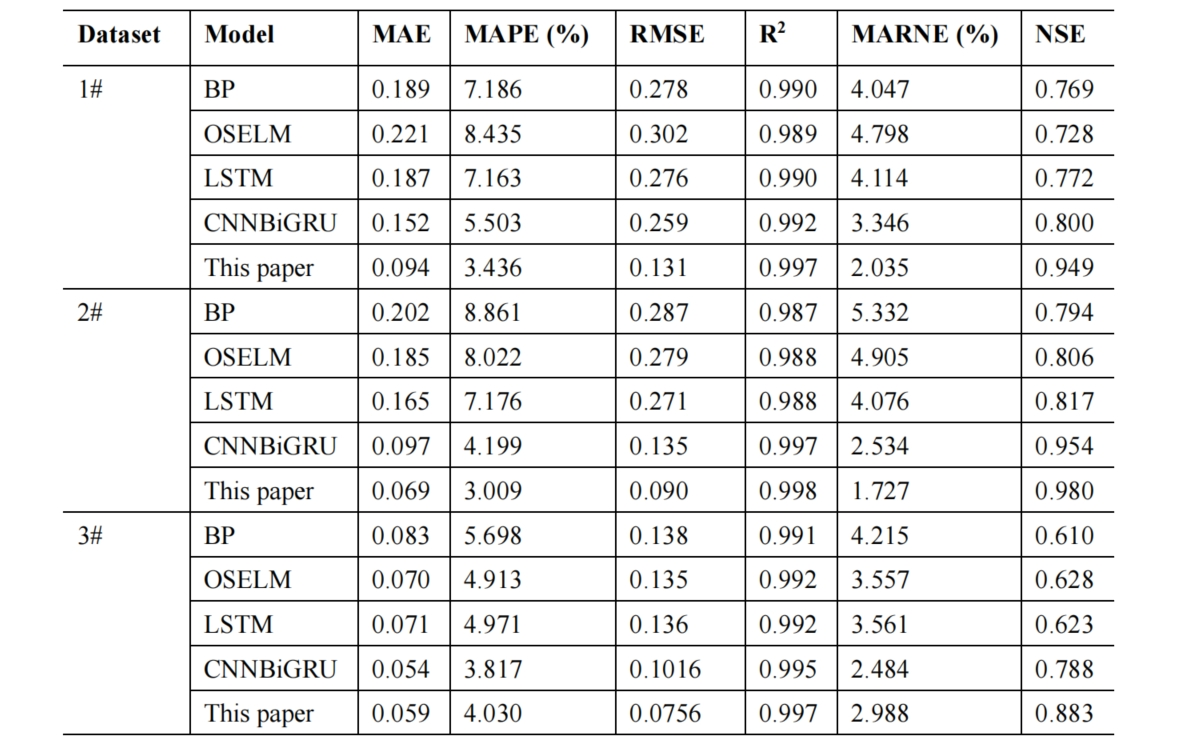

Supplement: S3 Table — (TIF) [file pone.0301349.s014.tif]

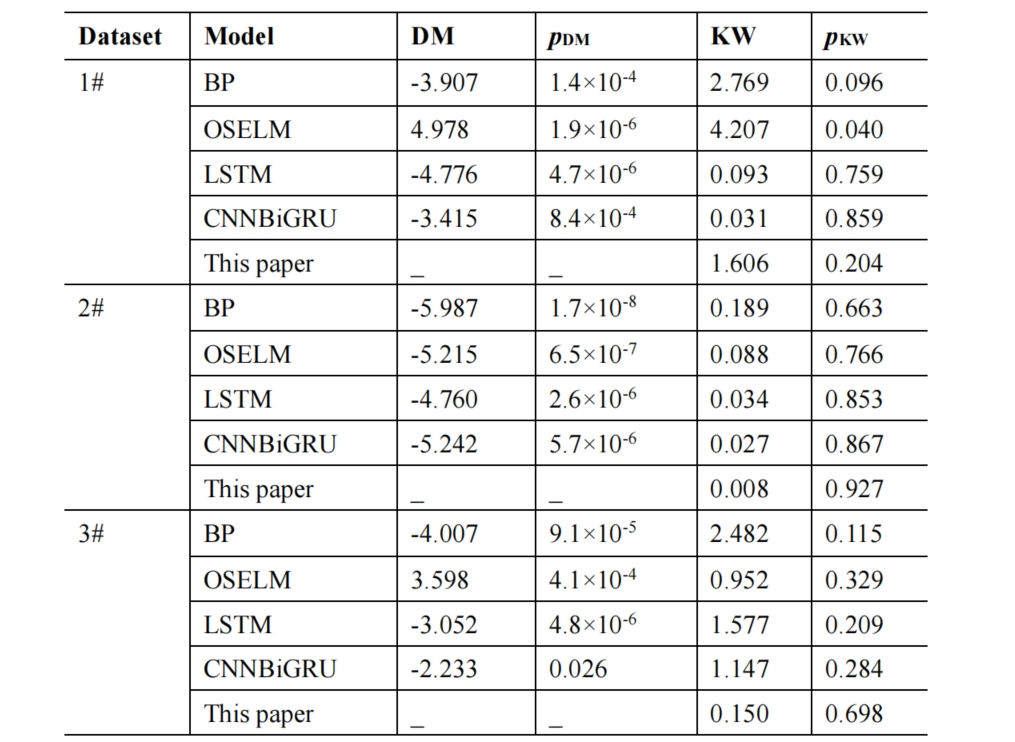

Supplement: S4 Table — (TIF) [file pone.0301349.s015.tif]
